# Per/Polyfluoroalkyl Substances (PFASs) in a Marine Apex Predator (White Shark, *Carcharodon carcharias*) in the Northwest Atlantic Ocean

**DOI:** 10.1021/acsenvironau.3c00055

**Published:** 2024-01-14

**Authors:** Jennifer Marciano, Lisa Crawford, Leenia Mukhopadhyay, Wesley Scott, Anne McElroy, Carrie McDonough

**Affiliations:** † Department of Civil Engineering, Stony Brook University, Stony Brook, New York 11794, United States; ‡ School of Marine and Atmospheric Sciences, Stony Brook University, Stony Brook, New York 11794, United States; § Department of Chemistry, Carnegie Mellon University, Pittsburgh, Pennsylvania 15213, United States

**Keywords:** PFAS, PFTrDA, PFPeDA, bioaccumulation, biomagnification, HRMS, white shark

## Abstract

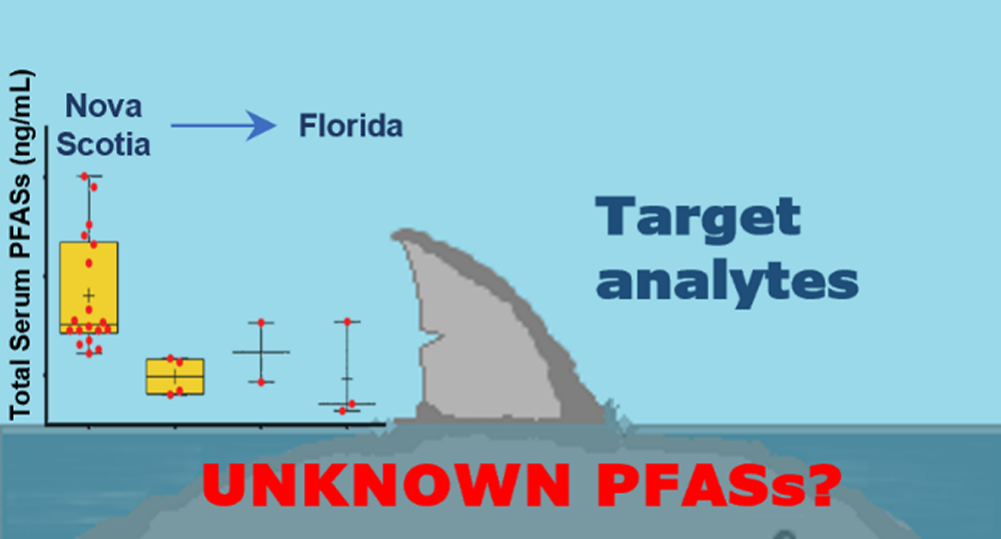

Per/polyfluoroalkyl substances (PFASs) are ubiquitous, highly persistent anthropogenic chemicals that bioaccumulate and biomagnify in aquatic food webs and are associated with adverse health effects, including liver and kidney diseases, cancers, and immunosuppression. We investigated the accumulation of PFASs in a marine apex predator, the white shark (*Carcharodon carcharias*). Muscle (*N* = 12) and blood plasma (*N* = 27) samples were collected from 27 sharks during 2018–2021 OCEARCH expeditions along the eastern coast of North America from Nova Scotia to Florida. Samples were analyzed for 47 (plasma) and 43 (muscle) targeted PFASs and screened for >2600 known and novel PFASs using liquid chromatography coupled to high-resolution mass spectrometry (LC-HRMS). Perfluoroalkyl carboxylates with carbon chain-length C11 to C14 were frequently detected above the method reporting limits in plasma samples, along with perfluorooctanesulfonate and perfluorodecanesulfonate. Perfluoropentadecanoate was also detected in 100% of plasma samples and concentrations were estimated semiquantitatively as no analytical standard was available. Total concentrations of frequently detected PFASs in plasma ranged from 0.56 to 2.9 ng mL^–1^ (median of 1.4 ng mL^–1^). In muscle tissue, nine targeted PFASs were frequently detected, with total concentration ranging from 0.20 to 0.84 ng g^–1^ ww. For all frequently detected PFASs, concentrations were greater in plasma than in muscle collected from the same organism. In both matrices, perfluorotridecanoic acid was the most abundant PFAS, consistent with several other studies. PFASs with similar chain-lengths correlated significantly among the plasma samples, suggesting similar sources. Total concentrations of PFASs in plasma were significantly greater in sharks sampled off of Nova Scotia than all sharks from other locations, potentially due to differences in diet. HRMS suspect screening tentatively identified 13 additional PFASs in plasma, though identification confidence was low, as no MS/MS fragmentation was collected due to low intensities. The widespread detection of long-chain PFASs in plasma and muscle of white sharks highlights the prevalence and potential biomagnification of these compounds in marine apex predators.

## 1 Introduction

Per/polyfluoroalkyl substances (PFASs) are a class of synthetic organic chemicals that are widespread in the environment and living things.^1−5^ They have been used in many consumer products such as nonstick cookware, personal care products, water- and stain-repellent clothing and furniture, as well as in aqueous fire-fighting foams (AFFFs), resulting in many pathways of environmental release and exposure.^6,7^ The stability of PFASs not only makes them desirable for consumer products but also highly persistent in the environment and capable of bioaccumulation.^1,8,9^ Elevated chronic exposure to several PFASs is associated with adverse health effects in humans and wildlife, including immunosuppression, thyroid dysregulation, and liver and kidney disease.^10−12^

Due to the widespread presence of PFASs in marine environments,^13^ marine organisms are susceptible to PFAS uptake, bioaccumulation, and toxic effects.^14,15^ Long-chain perfluoroalkyl acids (PFAAs) include perfluoroalkyl carboxylates (PFCAs) with carbon chain-length ≥8 (i.e., perfluorooctanoate (PFOA) and longer chain-lengths) and perfluoroalkyl sulfonates (PFSAs) with carbon chain-length ≥6 (i.e., perfluorohexanesulfonate (PFHxS) and longer chain-lengths). Long-chain PFAAs bioaccumulate due to their affinity for phospholipids and proteins like serum albumin and propensity for reabsorption.^9,16^ Several studies have also observed biomagnification of long-chain PFASs in aquatic food webs,^17−20^ though others have found inconsistent evidence of PFAA biomagnification.^20−22^ These discrepancies may be due to poorly understood tissue distributions of PFAAs, which do not accumulate in storage lipids like most legacy POPs,^9,23^ and/or poorly constrained contributions from PFAS precursors.^4,22,24^ Regardless, the widespread accumulation of PFASs in marine biota has been established^1,2^ and many studies have noted the predominance of long-chain PFCAs in tissues from fish, marine mammals, and other predators, including polar bears and birds.^18,20,21,25−28^ Increasing concentrations of long-chain PFCAs have been observed in marine biota, including Northern hemisphere mammals, fish, and birds.^29−31^

Along with legacy PFAAs like the PFCAs and PFSAs, novel PFASs have been detected in marine biota using high-resolution mass spectrometry (HRMS) techniques.^3,32,33^ A recent study observed significant contributions from unidentified organofluorine (30–75% of extractable organofluorine) in marine organisms in the Northwest Atlantic Ocean and identified 37 additional PFASs from 12 subclasses in tissue samples via HRMS suspect screening.^3^ HRMS suspect screening and nontarget analysis also led to the tentative identification of 54 PFASs in nine different subclasses in the livers of beluga whales from the St. Lawrence Estuary^32^ and 44 PFASs from nine different subclasses in livers collected from cetaceans in the South China Sea.^33^ Among these studies, ether-substituted PFSAs and PFCAs (PFESAs and PFECAs),^3,32^ unsaturated/cyclic PFSAs,^3,32,33^ hydrogen-substituted PFAAs and PFESAs,^32,33^ perfluoroalkyl sulfonamides (FASAs),^3,32,33^ and *x*:3 fluorotelomer carboxylates (*x*:3 FTCAs)^3,32,33^ were tentatively identified with varying levels of confidence. Despite many studies providing detailed HRMS analysis of PFAS accumulation in birds, fish, and marine mammals, this technology has not (to the best of our knowledge) been applied to the analysis of tissues or fluids from sharks.

White sharks are considered endangered according to the Committee on the Status of Endangered Wildlife in Canada^34^ and are particularly vulnerable to population declines because of their large size, long lives, and low abundance.^35^ Exposure to pollutants is a serious concern for the health of white sharks and other top predators due to biomagnification; studies have observed that white sharks and similar predators often accumulate greater levels of contaminants than lower trophic levels, including polychlorinated biphenyls (PCBs), polybrominated diphenyl ethers (PBDEs), dichlorodiphenyltrichloroethane (DDT), other organohalogens, and mercury.^36−40^ The shark’s lipid-rich liver is a major site of contaminant accumulation^38^ but many biomonitoring studies evaluate accumulation in muscle tissue because it is not as responsive to short-term changes in lipid content and is thus more representative of long-term exposures.^40^ Differences in bioaccumulation between white sharks and other species have been ascribed primarily to differences in diet, some of which may stem from different degrees of usage of inshore habitats and differences in metabolism and growth rate.^37,40^ Diet is considered the primary source of bioaccumulation of organic pollutants in sharks^37,38^ and concentrations have been observed to increase with size/age, though this relationship may be disrupted by growth dilution effects.^37,40^ Maternal offloading via mobilization of lipids from the liver during vitellogenesis has also been identified as a source of lipophilic organohalogens in young-of-year white sharks.^37,41^

Studies have observed bioaccumulation of PFASs in multiple shark species, including basking, blue, tiger, and bull sharks.^42−46^ The accumulation of odd-numbered long-chain PFCAs is commonly observed in fish and is, in part, attributed to atmospheric degradation of gas-phase fluorotelomer alcohols (FTOHs).^47^ Cartilaginous fish (sharks and rays) exhibit a distinct PFAS accumulation profile compared to bony fish, with PFCAs of length ≥C11 (particularly C11 (PFUdA), C13 (PFTrDA), and C14 (PFTeDA)) present at greater concentrations than in bony fish.^1^ PFUdA, PFTrDA, and PFTeDA were frequently detected (>95% of samples) in muscle from bull sharks and tiger sharks in the Southwest Indian Ocean, with total PFCA concentrations consistently greater than PFOS.^45^

The goal of this study was to evaluate the accumulation of PFAS in white sharks (*Carcharodon carcharias*) in the Northwest Atlantic Ocean. We used liquid chromatography and high-resolution mass spectrometry (LC-HRMS) to measure concentrations of 47 PFASs in shark plasma and muscle tissue and screen for >2000 additional PFASs in shark plasma from 27 white sharks in the Northwest Atlantic Ocean (Figure S1). Our objectives were to (1) provide baseline occurrence data on concentrations of PFASs in white sharks, (2) evaluate geographical differences in PFAS body burden and contaminant profiles, and (3) determine the extent to which LC-amenable novel PFASs beyond our target analytes contributed to PFAS burden in these apex predators.

## 2 Materials and Methods

### 2.1 Sample Collection and Preparation

#### 2.1.1 Sample Collection

White sharks (*N* = 27) were captured and sampled on the M/V OCEARCH off the coasts of Nova Scotia (2019 and 2020), Massachusetts (2020), North and South Carolina (2021), and Florida (2019) by the OCEARCH research team (Figure S1). Protocols were reviewed and approved by Jacksonville University’s Institutional Animal Care and Use Committee (IACUC). Protocols for capture and sampling are described in detail by Crawford et al. and Franks et al.^36,48^ Briefly, the sharks were brought onboard the M/V OCEARCH research vessel on a hydraulic platform and ventilated with seawater from a high-pressure hose.^48^ Blood samples were taken via caudal venipuncture and transferred by syringe to a lithium-heparin-coated vacutainer, inverted, and then centrifuged for 10 min at 3000 rpm to separate plasma from red blood cells. Muscle tissue was removed by using a surgical scoop after making a 2 cm incision below the first or second dorsal fin. Morphometric data and sex were recorded for each shark (Table S1) and life stage was assigned based on total length as described by Franks et al.^48^ Males were classified as juveniles (<2.80 m total length (TL)), subadults (2.81–3.45 m), and adults (>3.46 m) and females as juveniles (<3.30 m), subadults (3.31–3.79 m), maturing (3.80–4.19 m), and mature (>4.20 m), where the difference between “maturing” and “mature” depended on size in comparison to estradiol levels.^36^ Plasma samples were collected from 27 sharks, and muscle samples were collected from a subset of 12 sharks in Nova Scotia and Massachusetts. All biological samples were stored at −20 °C onboard and then transported to the laboratory, where they were stored at −80 °C until preparation and analysis.

#### 2.1.2 Sample Preparation

100 μL aliquots of plasma were transferred to Agilent Captiva enhanced matrix removal (EMR)-lipid cartridges with 1 ng of mass-labeled PFAS internal standards. Crash solvent (0.1 M formic acid in acetonitrile stored at −20 °C overnight) was added and left to sit for 10 min for protein denaturation and precipitation. The cartridges were then eluted into polypropylene autosampler vials using a positive pressure manifold and ultrahigh-purity nitrogen at a rate of 3–5 drops/min. Mass-labeled PFOA injection standard (1 ng) was added to each autosampler vial, and autosampler vials were capped, vortexed, and stored at −20 °C until analysis.

Muscle samples were weighed and transferred to 2 mL bead mill tubes containing ceramic beads, then diluted 3:1 with Optima LCMS-grade water and homogenized on a bead mill for 4 min at 120 ms^–1^. Some samples remained too thick to aliquot after homogenization, so additional water was added in 20 μL increments and rehomogenized until the slurry could be consistently drawn up. The final dilution factors for each sample were recorded (Table S6). 200 μL aliquots were transferred to a 2 mL polypropylene microcentrifuge tube, and 0.1 ng of mass-labeled internal standard was added. The mixture was diluted 4:1 with crash solvent, and samples were vortexed for 30 s, then centrifuged at 4 °C and 8400*g* for 5 min. Supernatants were passed through poly(ether sulfone) (PES) syringe filters (0.2 μm pore size) into 2 mL polypropylene autosampler vials, followed by 100 μL of methanol to rinse the syringe filter. Roughly 0.25 mg of Envi-Carb was then added, and samples were vortexed for 30 s and then centrifuged at 16,800 × *g* for 5 min. The supernatants were transferred to new autosampler vials, concentrated under nitrogen at 40 °C until just dry, and then reconstituted with 100 μL of 1:1 2 mM ammonium acetate in Optima water and acetonitrile containing 0.1 ng of mass-labeled PFOA injection standard. The samples were transferred into 250 μL vial inserts, capped, and stored at −20 °C until analysis.

### 2.2 Sample Analysis

Samples were analyzed on an Agilent 1290 Infinity liquid chromatograph coupled to an Agilent 6545 quadrupole-time-of-flight mass spectrometer (LC-QTOF-MS) to measure concentrations of target PFASs and simultaneously screen for novel PFASs. The chromatographic method used was adapted from McDonough et al.^49^ Briefly, 20 mM ammonium acetate in Optima LCMS-grade water and acetonitrile were used as the aqueous (A) and organic (B) mobile phases, respectively. The gradient started at 5% B and reached 100% B by 13.5 min with a flow rate 0.4 mL min^–1^. Samples were injected (35 μL plasma extracts, 30 μL tissue extracts) onto an Agilent Poroshell 120 EC-C18 analytical column (3 × 100 mm × 4 μm) preceded by a Phenomenex Gemini C18 guard cartridge (4 × 2.0 mm I.D.) and two Agilent Zorbax Diol guard cartridges (4.6 mm × 12.5 mm × 6 μm) in series. A delay column (Agilent InfinityLab PFC Delay Column, 4.6 × 30 mm) was installed in the binary pump after the solvent mixer to prevent potential interference of background PFASs in the mobile phase. The detector was operated in negative electrospray ionization (ESI^–^) mode (additional source parameters are in Table S2). Acquisition was done in All Ions Mode (data independent acquisition; DIA) with three collision energies (0, 10, and 35 eV) to screen for all mass-to-charge (*m*/*z*) ratios from 100–1200 Da.

#### 2.2.1 Targeted Quantitation

Calibration curves were prepared for >40 target analytes listed in Tables S3 (plasma) and Table S4 (muscle). Branched PFOS isomers (br-PFOS) were integrated as a sum of all detected isomers, and concentrations were estimated based on the response factor for the summed branched isomers in the PFOS analytical standard. In cases where branched isomers of other compounds were detected (e.g., PFHxS), they were included in the total peak area for linear and branched isomers, and concentrations were determined based on the response factor for the linear standard. Calibration curves were required to have at least four successive points with calculated concentrations within ±30% of expected values and *r*^2^ ≥ 0.95 with 1/*x* weighting. For plasma analysis, a matrix-matched curve ranging from 0.02–40 ng mL^–1^ plasma was used. Each calibration point was prepared according to the plasma preparation protocol using bovine calf serum as the matrix. Continuing calibration verifications (CCVs) were also performed in bovine calf serum at concentrations of 2 ng mL^–1^. The calibration curve for muscle was not matrix-matched due to the unavailability of blank tissue and was instead prepared in a solvent with the same composition as the samples. The calibration range corresponded to 0.43–100 pg g^–1^ ww tissue. CCVs were not prepared alongside the muscle calibration curve due to the low number of samples (*N* = 12); however, variability in the abundance of each mass-labeled internal standard was monitored and fairly consistent in all samples (<50% RSD), suggesting it was unlikely there were major changes in instrument accuracy over this short runtime.

Method reporting limits (MRLs) were determined by using the greater value of either the average blank concentration plus 3× the standard deviation of the blanks or the concentration of the lowest calibration point included in the calibration curve. MRLs are listed in Table S3 (plasma MRLs 0.04–0.90 ng mL^–1^) and Table S4 (muscle MRLs 1–42 pg g^–1^ ww). Concentrations < MRL were replaced with MRL/√2 as recommended for small sample numbers by Tekindal et al.^50^ Concentrations below the detection limits of the instrument (i.e., no peak observed above signal:noise level of 3) were replaced with zero.

#### 2.2.2 HRMS Suspect Screening

Suspect screening was completed via batch targeted feature extraction in Agilent Profinder using an in-house extracted ion chromatogram (XIC) list of >2000 PFASs that was adapted from the NIST Suspect List of Possible PFAS.^51^ Annotations were filtered based on mass accuracy (mass error within ±5 ppm), isotope abundance (≥2 isotopes detected), and peak height (≥1000 counts). Any peak areas ≤10*x* the average peak area in the blanks were replaced with zero. Remaining data were also filtered by retention time (≤ ±4 min away from expected retention time based on the RT vs *m*/*z* relationship defined for PFAS target analytes).

After these filtering steps, additional manual curation was completed to evaluate annotations. Several fluorotelomer alcohol-based compounds (FTOHs) were detected, which was surprising as these compounds do not ionize well by ESI^–^ and we would not expect our analysis to be very sensitive for these types of compounds. We confirmed this by injecting 6:2 FTOH onto the instrument at an expected mass of 0.5 ng on-column, for which we observed no signal above noise. For this reason, all FTOH-based compounds were removed from the list of identifications. We also omitted all PFASs identified for which detection frequency was >90% and peak area was consistent (within 20%) among all samples, as this suggests a signal that is likely due to endogenous compounds or other interferences such as shipboard background. To collect data-dependent fragmentation data for compounds of interest using limited volume samples, composite samples (one per location) were made using equal parts of multiple plasma samples to achieve sufficient volume (100 μL) and were prepared for analysis as previously described. They were analyzed via the same method except with data-dependent acquisition (DDA) to collect fragmentation of the specified ions (*m*/*z* for frequently detected novel PFASs were targeted). Confidence levels of annotations were assigned based on the PFAS Confidence Scale.^52^ To obtain estimates of relative abundance for tentatively identified novel PFASs with no analytical standards, peak areas were normalized using the mass-labeled internal standard with the closest retention time (Table S8).^49,53^ Semiquantitative concentration estimates were provided for perfluoropentadecanoate (PFPeDA) in plasma and muscle by inheriting the response factors for PFTeDA.

#### 2.2.3 Quality Assurance and Quality Control

Blank bovine serum (*N* = 5) and Optima LCMS-grade water samples (*N* = 3) were prepared alongside the plasma samples. Due to trace background PFAS contamination in the bovine calf serum stock, the solvent blanks were considered more representative of potential laboratory-based interferences and were used for blank censoring and MRL calculations. All average blank levels fell below the lowest included calibration point with the exception of chlorinated PFOS (Cl-PFOS, MRL = 0.08 ng mL^–1^). LCMS-grade water samples (*N* = 3) were also prepared alongside the muscle samples. Two of these blanks were used for blank filtering and MRL determination due to contamination of the third blank by native standard.

Matrix samples fortified with known amounts of native PFASs were prepared alongside both plasma and muscle samples. For plasma analysis, bovine calf serum samples fortified with native analytes at 2 ng mL^–1^ (*N* = 5) were prepared. As no blank muscle tissue sample was available, three muscle samples were chosen at random for fortification with 0.07 ng of native PFASs. The known (from previous analysis) concentrations of each compound in the nonfortified muscle samples were subtracted from the total to calculate analyte recoveries. The average recoveries for all PFASs in fortified plasma were within ±30% of the expected value (Figure S2). The average recoveries in fortified muscle samples were within ±30% for 32 compounds, <70% for eight compounds, and >130% for two compounds (Figure S3).

### 2.3 Statistics and Data Analysis

Box plots were created by using GraphPad Prism 6.0. Correlation matrices were created using R (package “corrplot”).^54,55^ Only frequently detected (≥50% detection frequency) compounds were included in statistical analyses and figures, with concentrations <MRL replaced by MRL/sqrt(2) and concentrations <DL replaced with 0. Group comparisons (e.g., sharks from different locations) were compared using one-way ANOVA in Excel. For all statistical tests, significance was defined as *p* ≤ 0.05.

## 3 Results and Discussion

### 3.1 PFASs in Shark Plasma and Muscle Tissue

**Figure 1:**
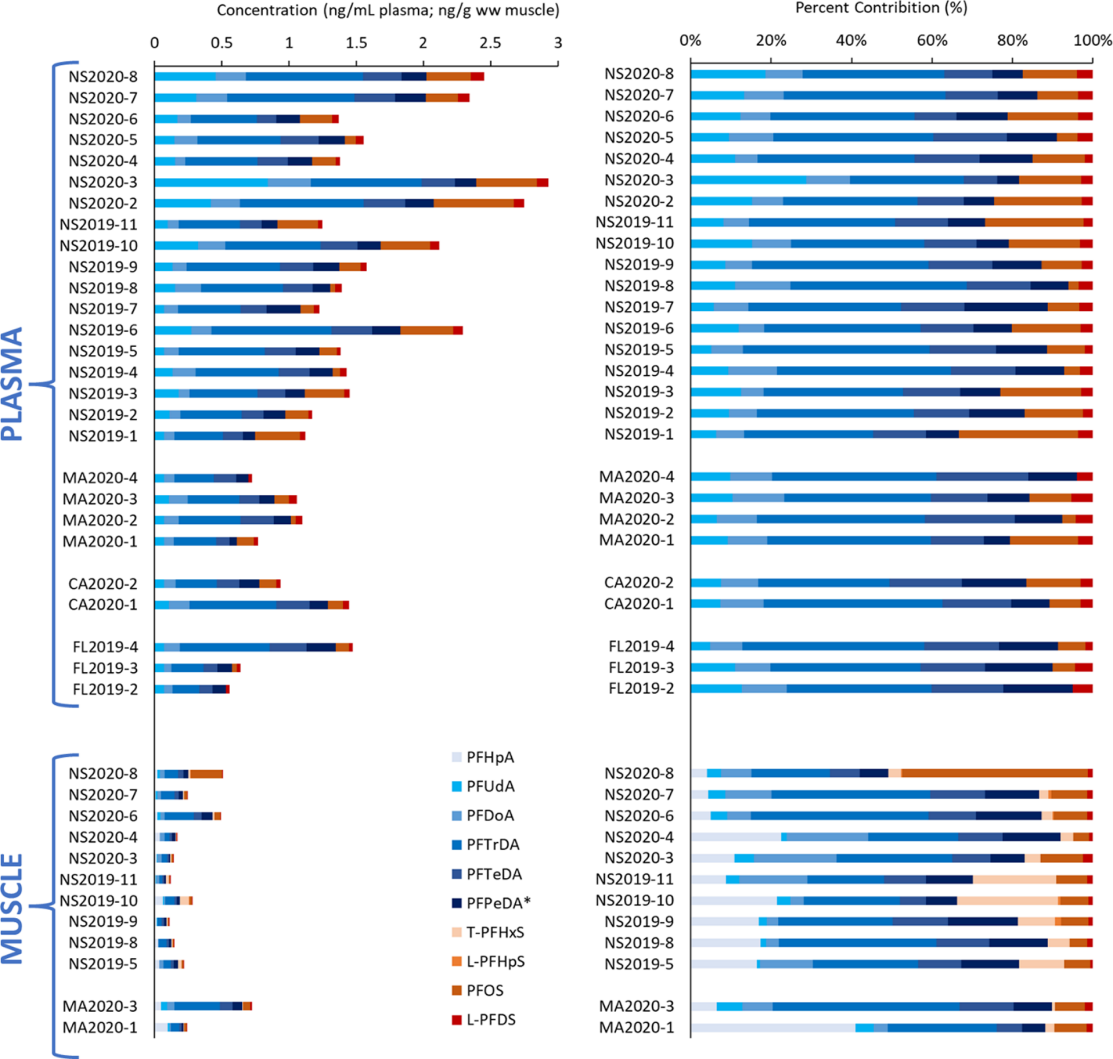
PFAS concentrations in white shark plasma and muscle. Concentrations of frequently detected (≥50% detection) PFASs in plasma samples (ng mL^–1^) and muscle tissue samples (ng g^–1^ ww) from off the coasts of Nova Scotia (NS), Massachusetts (MA), the Carolinas (CA), and Florida (FL). Concentrations of PFPeDA were estimated semiquantitatively using inherited response factors for PFTeDA.

Eight long-chain PFAAs (C8–C16; chain-length referring to total number of carbons) were detected >MRL via targeted analysis in plasma samples (all concentrations in Table S5). Of these, six PFAAs (C11–C14 PFCAs, linear L-PFOS, and L-PFDS) were detected frequently (≥50% detection; Figure 1) while PFHxDA and summed br-PFOS were detected >MRL in one and three samples, respectively. Several other PFASs (PFNA, PFDA, L-PFHpS, L-PFDoS, and chloroperfluorooxaundecanesulfonic acid (Cl-PFOUdS)) were detected > DL but < MRL (i.e., peaks were observed above 3:1 signal:noise but concentrations could not be determined) in multiple samples and are listed in Table S5. PFPeDA was identified via HRMS and detected in all plasma samples, displaying a consistent RT vs *m*/*z* trend with the PFCA homologous series (confidence level 2c).^52^ An analytical standard was not available for PFPeDA, so concentrations were estimated based on response factors for PFTeDA.

A greater number of PFASs (16) were detected in muscle tissue, including C6–C8 and C10 PFSAs, C5 and C7–C14 PFCAs, and perfluorooctane sulfonamide (FOSA), with nine PFASs (all PFCAs and PFSAs) detected in >50% of the samples (Figure 1). All detected PFASs were long-chain PFAAs except for the C5 and C7 PFCAs (PFPeA and PFHpA). Detection of short-chain PFCAs in biota is somewhat unexpected as they are excreted relatively easily in comparison to other PFCAs,^56,57^ but the use of HRMS and rejection of peaks with mass error >±5 ppm prevents common false positives that might occur due to endogenous interferences.^58^ PFHxS (primarily linear with minor contributions from branched isomers) also appeared to contribute to PFAS burden in muscle, though it was not detected in plasma. These compounds were not reported in any of the other available published studies of PFASs in other shark species, and their detection in plasma and muscle of white sharks warrants further research. Muscle-plasma ratios calculated by dividing the concentration of each compound in muscle (ng g^–1^ ww) by the concentration in serum (ng mL^–1^) were all <1, indicating a greater affinity of these compounds for plasma. This has also been observed in studies of PFAS tissue distributions in fish^59^ due to the high affinity of PFASs for transporter proteins like serum albumin.^60^

The sum of the seven frequently detected PFASs in plasma, including PFPeDA (Σ_7_PFAS) ranged from 0.56 ng mL^–1^ in a female subadult shark on the Florida coast (FL2019-2) to 2.93 ng mL^–1^ in a male subadult shark off of Nova Scotia (NS2020-3), with a median of 1.38 ng mL^–1^. PFAS percent composition was fairly consistent among all plasma samples from all locations (Figure 1). In muscle, ∑_9_PFAS (sum of 9 PFASs detected in ≥50% of samples) ranged from 0.20 ng g^–1^ ww in an adult male collected of the coast of Nova Scotia (NS2019-09) to 0.84 ng g^–1^ ww in a juvenile male collected off the coast of Massachusetts (MA2020-03). No correlation between muscle and plasma levels was readily apparent for the subset of 12 individuals for which paired samples were collected. PFTrDA was typically most abundant in both plasma and muscle, contributing 27–44% (median 36%) of Σ_7_PFAS in plasma and 18–41% (median 23%) of Σ_9_PFAS in muscle. The prevalence of PFTrDA along with other odd-numbered long-chain PFCAs has been observed in studies of other shark species^43−45^ and suggests significant contributions from atmospheric degradation of PFAS precursors, which has been identified as a source of odd-numbered long-chain PFCAs to remote marine environments.^47^

Differences in PFAS composition seen in muscle tissue and plasma may be due to toxicokinetic considerations. Elasmobranchs have distinct physiology from other groups of marine vertebrates, with unique metabolism and membrane composition and lipid-rich livers that aid in maintaining buoyancy.^61^ Muscle is often analyzed in studies assessing pollutant exposure among sharks because this tissue is relevant for human consumption and less subject to variability due to changes in lipid levels than the liver, making it more representative of longer-term exposures.^40^ Analysis of muscle here facilitates comparisons to other species. However, the limited amount of information available on tissue distributions of PFASs in sharks suggests muscle does not have the greatest PFAS concentration; liver, heart, and gills all typically exhibited greater concentrations of total PFASs than muscle.^43,44^ To our knowledge, there are no studies measuring PFASs in shark blood, serum, or plasma, and therefore, no comparison among species could be done for these samples. Total PFAS burdens in white sharks were generally lower than what has been measured in tissues from marine mammals in other studies, although these studies typically analyze liver tissue, prohibiting a direct comparison. Still, studies reporting liver PFAS concentrations for sharks are generally low compared to marine mammals.^43^ While sharks are similar to marine mammals analyzed in other studies in that they are apex marine predators, they have several important physiological differences that may contribute to differences in total PFAS burdens.^61^ Additionally, gill respiration is a distinct route of uptake and excretion, impacting levels and composition of PFASs in sharks and other fish compared to marine mammals.^62^

PFPeDA has been identified based on *m*/*z* and consistent RT in several studies analyzing PFASs in biota, including Greenland and Arctic marine biota.^28^ While detection frequencies of PFPeDA in these studies were high, the percent contribution of PFPeDA to total estimated PFCA burden (estimated using PFTeDA as a reference standard, as was done here) was typically lower (<1% of ΣPFCA in Greenland polar bears and ringed seals; <2% in German Bight harbor seals; <5% in Greenland killer whales)^24,63^ than for white shark plasma (13 ± 3.4% of Σ_5_PFCA) or muscle (13 ± 3.7% of Σ_7_PFCA) analyzed here. However, it is important to note that liver tissue was analyzed in these other studies (compared to plasma and muscle here), and few studies have probed PFPeDA tissue distributions, so these values are not easily comparable.^63^

**Figure 2:**
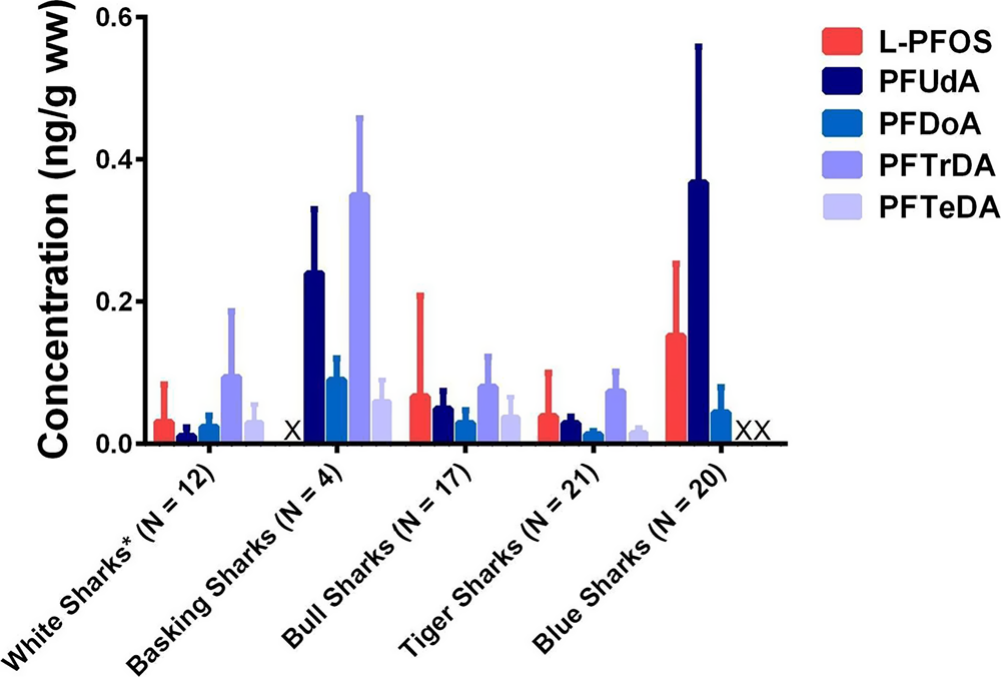
Comparison of average PFAS concentrations in muscle tissue between five different studies on a variety of species of sharks (basking sharks from the Mediterranean,^42^ bull sharks and tiger sharks from the Indian Ocean,^45^ and blue sharks from the eastern Atlantic Ocean^43^). “X” represents compounds that were not measured. Error bars represent the standard deviation in measurements. Concentrations used to produce this figure are provided in Table S7.

Total PFAS levels measured here are comparable to PFAS concentrations measured in other shark species in the majority of available studies (Figure 2), though there is not a lot of data to compare to. PFASs have been detected previously in shark tissues, including muscle and liver, and PFTrDA is typically the most abundant PFAS found in these tissues, consistent with this study.^44,45^ The concentrations of PFCAs in basking sharks (*Cetorhinus maximus*) in the Mediterranean Sea were similar in magnitude to those seen here, but somewhat greater.^42^ This was somewhat surprising, as basking sharks are filter feeders, and they may not be expected to be vulnerable to biomagnification. However, PFAS accumulation has been noted previously in both phytoplankton and zooplankton that are prominent in the diet of filter feeders.^64^ Tiger (*Galeocerdo cuvier*) and bull sharks (*Carcharhinus leucas*) feed at a similar trophic level as white sharks, and muscle samples collected from these species in the Indian Ocean exhibited concentrations that were fairly similar to those measured in our study.^45^ Muscle from blue sharks (*Prionace glauca*) in the Atlantic Ocean collected southwest of Portugal in 2016 had greater levels of average PFOS (0.152 ± 0.101 ng g^–1^ ww) and PFUdA (0.367 ± 0.191 ng g^–1^ ww) than were measured here, and PFTrDA was not measured.^43^

Another study was available for comparison but was not included in Figure 2 in the interest of clarity due to large differences in concentrations. Zafeiraki et al. investigated the presence of PFASs in eight shark species off the coast of Greece in the Aegean, Eastern Mediterranean, and Ionian Seas.^44^ All species of sharks sampled are apex predators and have trophic positions similar to those of white sharks. However, average total PFAS concentrations (Σ_10_PFAS) in shark muscle tissue were considerably greater than those in the current study, ranging from 1.6 ng g^–1^ ww in blue shark (*N* = 13) to 18 ng g^–1^ ww in angular roughshark (*N* = 1). This may be due in part to the method of collection: samples were collected from sharks for sale and consumption at local markets. Studies have observed greater PFAS concentrations in seafood sampled from markets as opposed to species sampled directly from the marine environment, potentially because many seafood products are captured near the coast.^65^ It is unclear whether the sharks collected in Zafeiraki et al.’s study originated close to point sources. If so, this could be a possible explanation for the differences in the total PFAS levels. While concentrations were elevated, the composition of PFASs was consistent with this and other studies, with PFTrDA generally most abundant.

#### 3.1.1 Geographical Differences in PFAS Body Burden

**Figure 3:**
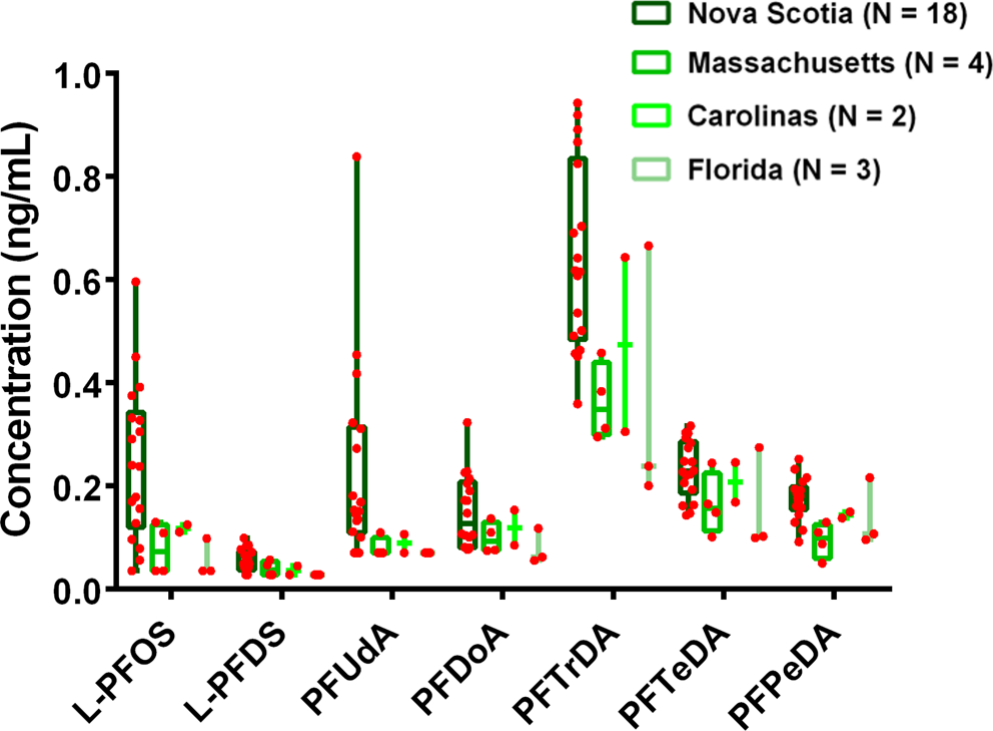
PFASs in shark plasma by location of collection. Distribution of seven frequently detected (>50% detection) PFASs in plasma for sharks from each sampling area, with number of samples collected noted in the legend. Boxes show the 25th to 75th percentile and the 50th percentile (median) value is marked by a horizontal line. Whiskers show the minimum and maximum range. Levels of all compounds were significantly (*p* < 0.05) greater in Nova Scotia samples than samples from other locations. Because *N* ≤ 3 for the Carolinas and Florida, only the median, minimum, and maximum are shown.

The maximum levels of all detected PFASs were measured in samples collected off the coast of Nova Scotia, but not all of the maximum levels were from the same shark. C12–C15 PFCAs were detected above reporting limits (0.04–0.07 ng mL^–1^) in all shark plasma collected from all locations, but detection of PFUdA and PFDS was dependent on sampling location, with greatest frequencies of detection in Nova Scotia. PFUdA was frequently detected in sharks sampled near Nova Scotia (83% detection) but was only found >MRL (0.10 ng mL^–1^) in two sharks from other locations. PFDS was detected >MRL (0.04 ng mL^–1^) more frequently (78% detection) in Nova Scotia samples compared to all other samples (33% detection). Levels of each frequently detected C12–C15 PFCA were significantly (*p* < 0.05) greater in Nova Scotia samples compared to all other samples (Figure 3). Plasma samples from Nova Scotia also had a wider distribution of Σ_7_PFAS than observed at other locations (Figure 3); however, sample sizes from other locations were small, and it was not readily apparent that the distribution of concentrations in Nova Scotia was due to any differences in population makeup such as age or size.

Several pairs of frequently detected PFASs displayed significant (*p* < 0.05) correlations among the plasma samples (Figure S4), suggesting common exposure sources. In general, PFCAs that were closer in chain-length tended to correlate more strongly (Figure S5). PFTeDA and PFTrDA correlated most strongly (*r*^2^ = 0.92; *p* = 2.9 × 10^–12^). PFOS correlated significantly with PFUdA and PFTrDA (*r*^2^ = 0.50–0.65; *p* < 0.05), but did not correlate strongly with the frequently detected even-chain PFCAs, PFDoA, and PFTeDA (*r*^2^ ≤ 0.33; *p* ≥ 0.08–0.15) or PFPeDA (*r*^2^ = 0.29; *p* = 0.13).

When samples from Nova Scotia were grouped compared to all other samples from off the coasts of Massachusetts, the Carolinas, and Florida, samples collected near Nova Scotia exhibited significantly greater Σ_7_PFAS levels than from other areas. Total plasma mercury concentrations were also greatest in samples from Nova Scotia.^36^ This was attributed to the fact that the sharks sampled from Nova Scotia tended to be larger (median weight, 450 kg) than those from the other three locations (median weight, 200 kg). However, in this study, we saw no significant correlation between shark size and PFAS burden. Nova Scotia was also the only location where sharks classified as adults were sampled (all sharks from the further south were juveniles or subadults). However, we found that concentrations were elevated in the Nova Scotia group compared to those in other locations regardless of whether adult sharks were included in the comparison, suggesting that age alone was not an explanation for this observation.

White sharks are a highly mobile species that annually migrate from northern regions near Massachusetts and Maritime Canada in the summer to the southeastern U.S. and the Gulf of Mexico in the winter.^35,48^ White shark movement is dependent on the animal’s size, sex, and maturity stage, as most individuals migrate along the continental shelf, while adult female sharks are capable of venturing hundreds of kilometers offshore.^48^ White sharks also display seasonal residency phases, with tagged individuals clustering near Cape Cod and Atlantic Canada for several months in the late summer and early autumn.^48^ Thus, white sharks sampled near Nova Scotia likely inhabited the region for a significant portion of their lives prior to sampling. Sharks sampled off the coast of Nova Scotia had also likely recently fed on pinnipeds.^36^ This may have resulted in significant recent exposure to bioaccumulative long-chain PFASs. Seasonal site fidelity near Nova Scotia may have contributed to repeated elevated exposure to PFASs via diet. Notably, fewer samples were available from off the coasts of Massachusetts, the Carolinas, and Florida, precluding a detailed analysis of geographical differences in PFAS plasma levels among samples from these other sites.

### 3.2 Additional PFASs Detected in Plasma via HRMS Suspect Screening

**Figure 4:**
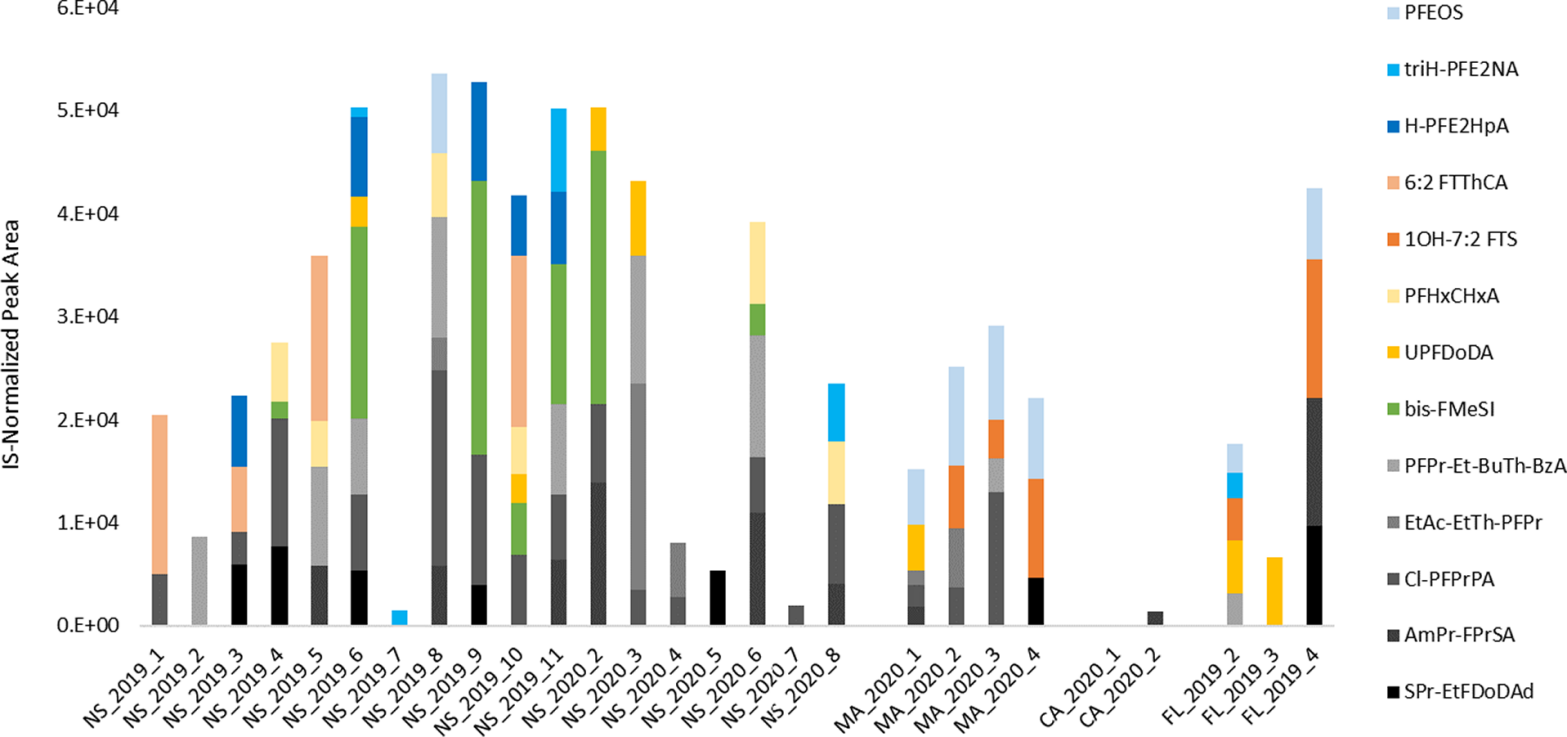
Compounds were tentatively identified at Confidence Level 4 in shark plasma samples via HRMS suspect screening. More information about annotations as well as full compound IUPAC names is provided in Table S8. Sample codes correspond to the location (NS: Nova Scotia; MA: Massachusetts; CA: Carolinas; and FL: Florida).

After manual curation and blank censoring, 13 novel PFASs (excluding PFPeDA) were tentatively identified via a HRMS suspect screening. None of these compounds were detected in serum blank samples or native spikes. Details on annotations, including average mass error and retention time, detection frequency, SMILES codes, average abundance, and confidence level, are provided in Table S8. Relative abundances after normalization are shown in Figure 4. All tentative identifications except for PFPeDA (for which identification confidence was supported by MS/MS fragmentation and PFCA homologous series) were assigned a confidence level of 4 based on exact mass and isotope pattern match,^52^ as MS/MS spectra were not collected during DDA runs due to low peak intensity. Due to the absence of fragmentation evidence, all of these tentative identifications can only provide an unequivocal molecular formula based on exact mass and isotope pattern and cannot differentiate between isomers, of which there are many for some of these structures.^52^

Due to the low confidence in novel PFAS identifications in this study, very little can be ascertained regarding accumulation or spatial trends on novel PFASs in white sharks from this limited sampling. It should be noted that there were geographical trends in the detection of novel PFASs, with distinct compositions observed in different regions and very few identifications in samples from off the coast of the Carolinas. Tentatively identified perfluoroheptane ether sulfonate (PFEOS; [C_7_F_15_SO_4_]^−^) was detected in all samples collected off of Massachusetts but only intermittently in other areas. As there are no other data available for HRMS suspect screening of novel PFASs in sharks, we compared our findings to some recent studies on other marine organisms, which have focused primarily on mammals.^3,32,33^ We found that a few of the compounds we tentatively identified have been detected in these other studies. PFEOS was detected by Spaan et al. in several species, including seals and whales, but fragmentation was not collected due to low intensity.^3^ Bis-FMeSI (bis(trifluoromethylsulfonyl)imide; [C_2_F_6_NO_4_S_2_]^−^) was tentatively identified primarily in Nova Scotia samples and was also previously detected by Wang et al. in seawater from the South China Sea.^33^ However, Wang et al. did not detect bis-FMeSI in marine biota via analysis of the whole body (crustaceans and fish) and liver tissue (mammals). Our detections of potential novel compounds in white shark plasma and their geographical distinctions could be linked to differences in diet, metabolism, and gill respiration, and the data highlight the need for further detailed analyses.

## 4 Conclusions

This study highlighted the accumulation of PFASs (particularly C11–C15 PFCAs) in plasma and muscle tissue of white sharks in the Northwest Atlantic Ocean and noted some geographical differences in PFAS body burdens that may be due to differences in the diet among these distinct shark populations. More PFASs, including some shorter-chain PFCAs, were detected in muscle tissue but not in plasma. Sharks off the coast of Nova Scotia exhibited significantly greater PFAS concentrations in plasma than samples collected from all other areas and a greater number of novel PFAS detections via HRMS suspect screening, though identifications were of low confidence due to insufficient signal to collect informative MS/MS fragmentation. PFTrDA was the most abundant PFAS in both muscle and plasma. The widespread detection of PFTrDA and PFPeDA highlights the prevalence of odd-numbered long-chain PFCAs (some of which are often not included on biomonitoring lists) in marine apex predators.

The detection of PFASs in marine biota far from point sources highlights the ubiquity of PFAS pollution. Our results suggest that diet is an important determinant for PFAS burdens in marine predators, although there remain many unknowns related to how differences in physiological makeup and metabolism impact PFAS accumulation and distribution in tissues of marine fish and mammals. A major limitation of this study was the small sample size of white sharks sampled from Massachusetts, the Carolinas, and Florida, as well as the low intensity of tentatively identified novel PFAS peaks, resulting in the lack of fragmentation data for HRMS suspect screening. These are common roadblocks to developing an understanding of pollutant impacts on marine biota in remote locations. By amassing data from limited studies like ours, future studies may be able to draw further conclusions about the impacts of PFASs and other contaminants on elusive apex predators such as the white shark.

## Supporting Information Available

Locations and dates of where the sharks were sampled for the current study, the average recoveries of the target analytes in the five native spike plasma samples, the average spiked muscle sample recoveries, correlation matrix for PFASs in plasma, and linear regression between the difference in chain-lengths of PFAAs and correlation coefficients for the pairs (PDF)
Shark metadata, QTOF detector parameters, calibration curve details, all quantitative data target and summary of nontarget data, and data used in literature comparison (XLSX)
