# Supplementary material for: Per/Polyfluoroalkyl Substances (PFASs) in a Marine Apex Predator (White Shark, Carcharodon carcharias) in the Northwest Atlantic Ocean
Source: ACS Environ Au. 2024 Jan 14;4(3):152–61. doi: 10.1021/acsenvironau.3c00055 (PMC11100321; doi:10.1021/acsenvironau.3c00055)
Supplement: Supplementary file 1 — vg3c00055_si_001.pdf [file vg3c00055_si_001.pdf]

SUPPORTING INFORMATION

Per/polyfluoroalkyl substances (PFASs) in a marine apex predator (white shark, *Carcharodon carcharias*) in the Northwest Atlantic Ocean

Jennifer Marciano[1]; Lisa Crawford[2]; Leenia Mukhopadhyay[3]; Wesley Scott[3]; Anne McElroy[2]; Carrie McDonough*[3]

[1] Stony Brook University Department of Civil Engineering, Stony Brook, NY 11794 USA
[2] Stony Brook University School of Marine and Atmospheric Sciences, Stony Brook, NY 11794 USA
[3]Carnegie Mellon University Department of Chemistry, Pittsburgh, PA 15213 USA

*Corresponding author: Carrie McDonough cmcdonou@andrew.cmu.edu

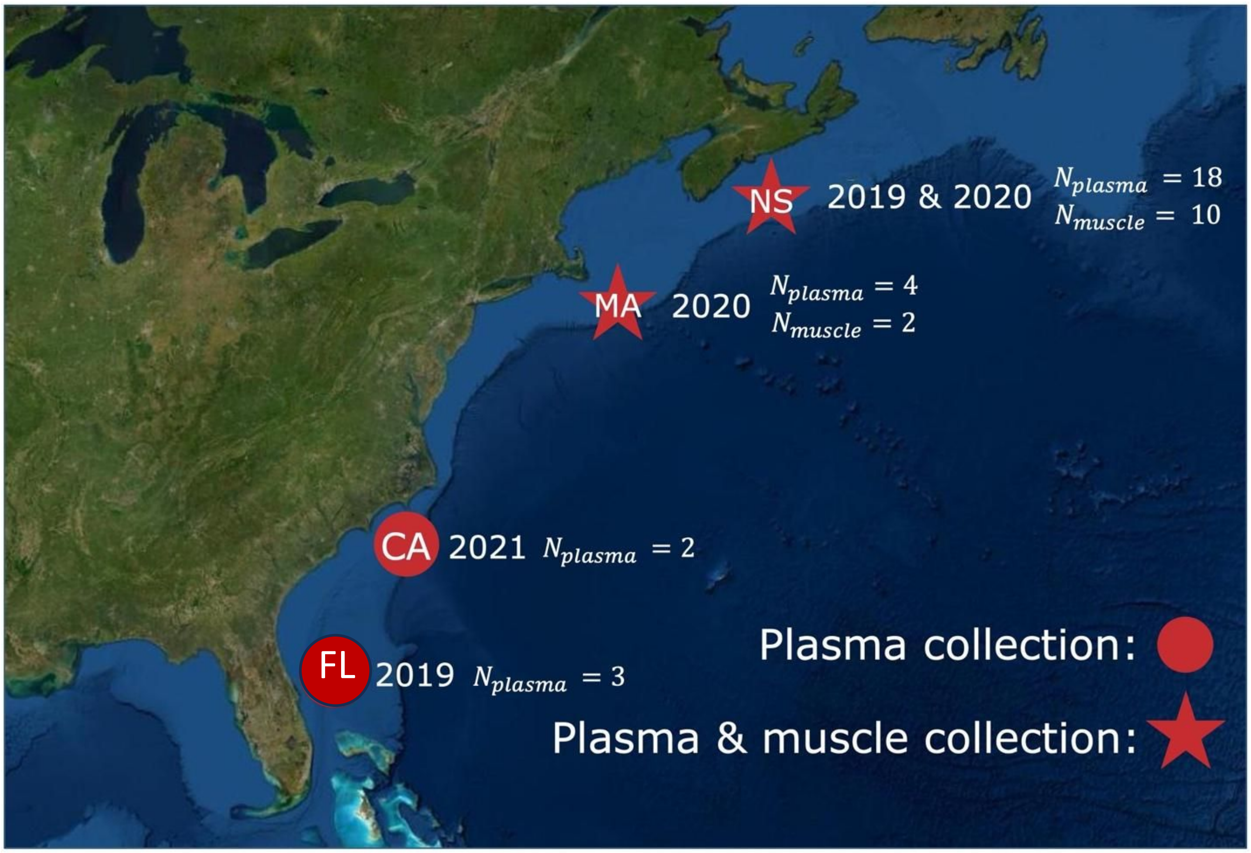

Figure S1: The locations and dates of where the sharks were sampled for the current study, where NS is Nova Scotia, MA is Massachusetts, CA is the Carolinas, and FL is the Southeast Coast of the United States (Florida). The number of plasma (N_{plasma}) and muscle (N_{muscle}) samples that were analyzed from each location is listed.

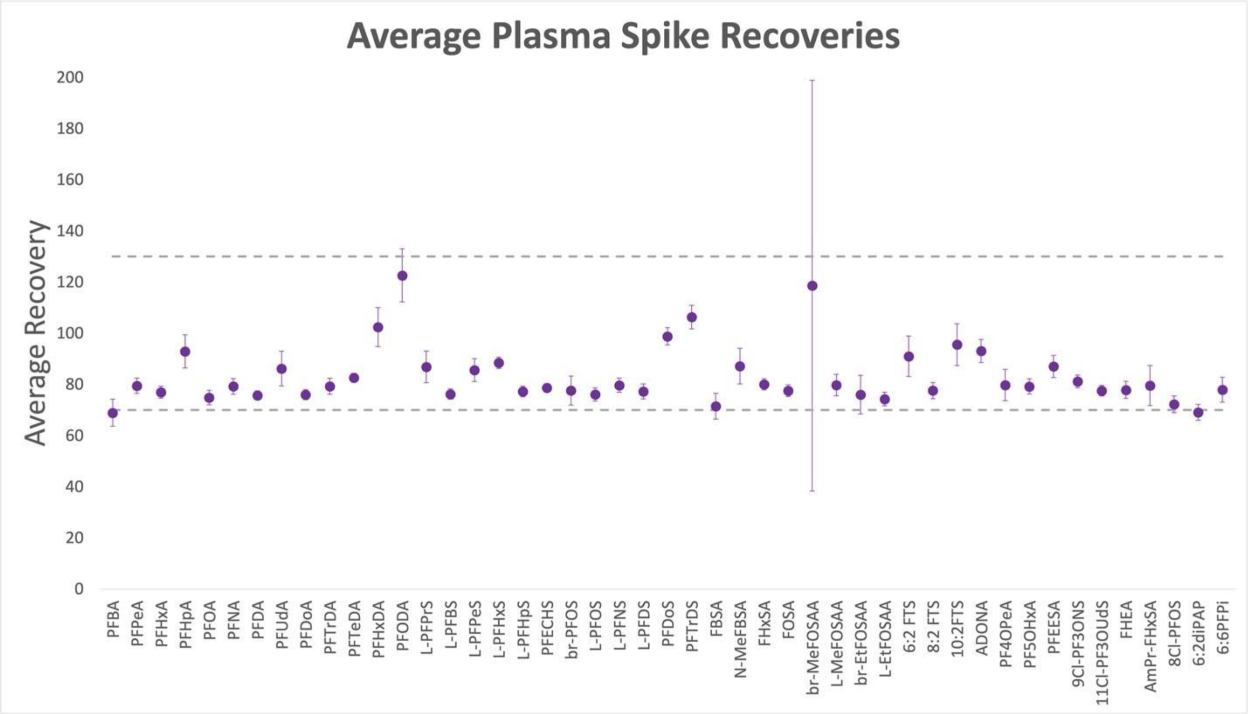

Figure S2: The average recoveries of the target analytes in the five native spike plasma samples.

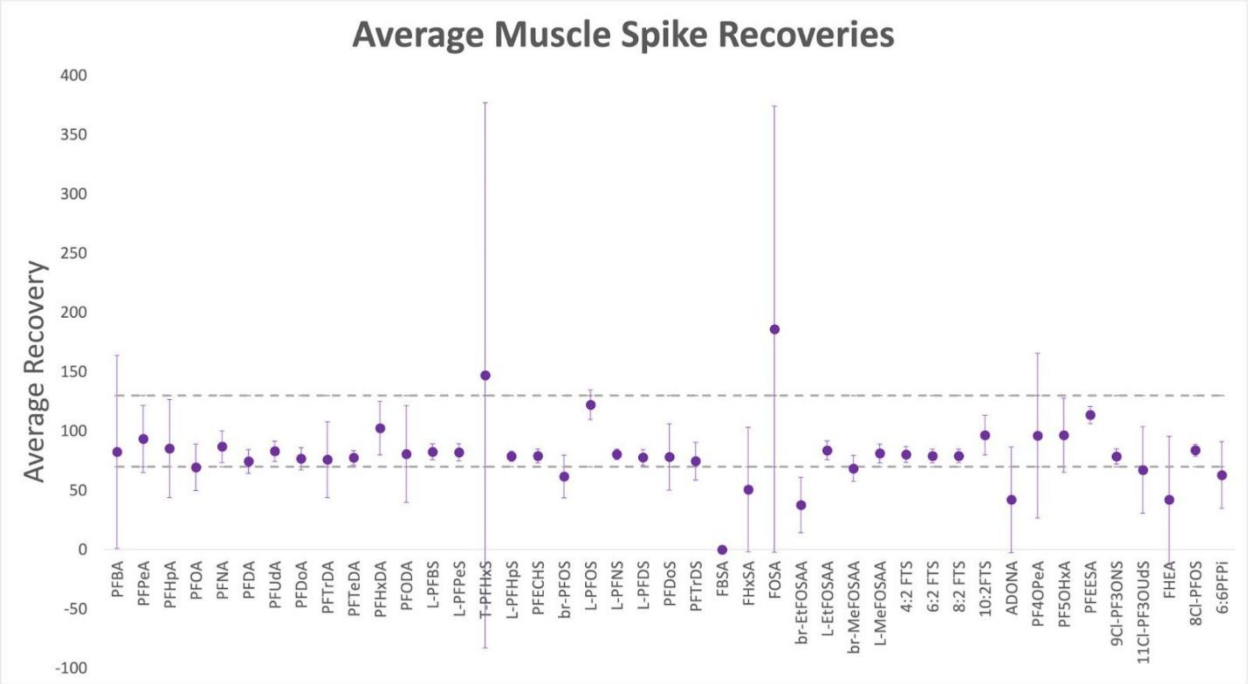

Figure S3: The average spiked muscle sample recoveries.

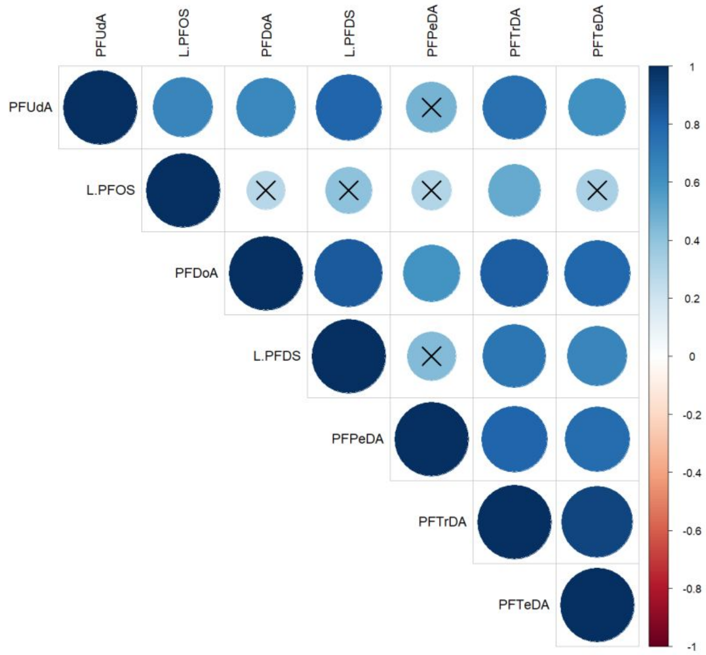

Figure S4: Correlation matrix for PFASs in plasma. Positive correlations are displayed in blue, and negative correlations (of which there are none) are displayed in red. The size of the circle is related to the Spearman's rank correlation coefficient (R_s). Larger circles denote stronger correlations.

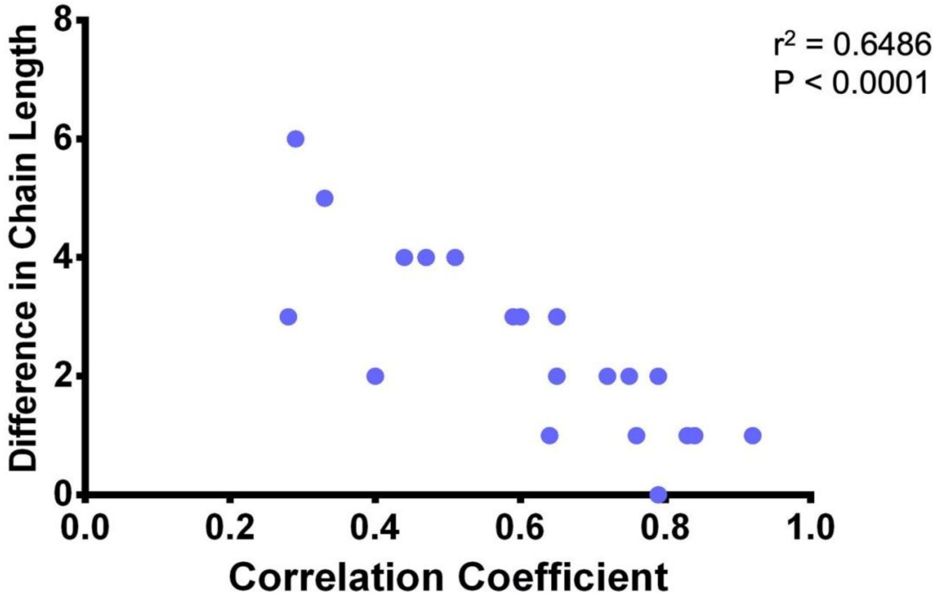

Figure S5: Linear regression between the difference in chain-lengths of PFAAs and correlation coefficients for the pairs listed in **Figure S4**.
